# Treatment of neuropathic pain with repeated low-dose MDMA: a case report

**DOI:** 10.3389/fpsyt.2025.1513022

**Published:** 2025-02-03

**Authors:** Peter Gasser, Matthias E. Liechti, Friederike Holze

**Affiliations:** ^1^ Private Practitioner, Solothurn, Switzerland; ^2^ Clinical Pharmacology and Toxicology, University Hospital Basel and University of Basel, Basel, Switzerland

**Keywords:** MDMA, LSD, microdosing, chronic pain, limited medical use, psychedelic-assisted therapy

## Abstract

A 64-year-old male patient who suffered from traumatic life experiences and neuropathic pain after oncological chemotherapy was treated with medium to high doses of lysergic acid diethylamide (LSD) and high doses and microdoses of methylenedioxymethamphetamine (MDMA). At the beginning of treatment, the patient did not experience any acute subjective effects of LSD at a dose of 200 µg. After increasing the LSD dose to 400 µg, he experienced subjective acute effects, and the first lasting therapeutic effects were observed. After changing from LSD to MDMA at both high doses (150-175 mg) and repeated low doses (12.5-25 mg), the patient exhibited marked improvements in neuropathic pain that were sustained even after stopping repeated MDMA treatment. MDMA mini/microdosing has not yet been broadly investigated. This case documents benefits of low doses of MDMA for the treatment of a pain disorder. Further research is needed on effects of MDMA on pain.

## Background

Since the early 1960s, the analgesic efficacy of classic psychedelics, which act via agonism on serotonin 2A receptors, in the treatment of cancer patients has been questioned (1). Experimental results have been inconclusive, and treatment with psychedelics has subsequently focused on psychological disorders and coping with life-threatening and sometimes painful illnesses (2–5). However, Goel et al. (6) reported evidence of an analgesic effect of psychedelics on tumor-related pain and certain forms of headache. Methylenedioxymethamphetamine (MDMA), which is not considered a classic psychedelic, acts as monoamine and oxytocin releasing agent (7, 8). It has shown only limited evidence of an analgesic effect that goes beyond more immediate effects of the substance (9). In a study in patients with posttraumatic stress disorder, MDMA reduced chronic pain intensity and disability in a subgroup of patients with the highest chronic pain scores (9). Our own clinical research and experiences have led us to suspect that acute effects of psychedelics can lead to pain relief through general relaxation and focusing on other areas of experience, but they can also lead to pain intensification through a stronger focus on the pain itself. Among classic psychedelics, lysergic acid diethylamide (LSD) and psilocybin have shown a pain-mitigating effect through microdosing (i.e., the intake of low doses with minimal acute psychedelic effects every few days over several weeks or months), which has been reported in individual case reports or anecdotal reports (10). We found no related published reports for MDMA. In the present case, we observed improvements of a neuropathic pain disorder with both high doses (150-175 mg) and low doses (12.5-25 mg) of MDMA.

## Case presentation

The male patient, born in 1960, was 58-64 years old during the course of treatment. He grew up as the older of two sons in a rural area of Switzerland in a middle-class family. He and his brother suffered from severe physical abuse at the hands of their father throughout childhood and adolescence, and they were not protected by their mother, who did not intervene. During adolescence, the patient experienced a severe psychological crisis with the temporary excessive consumption of legal and illegal substances of abuse (alcohol, tobacco, marihuana, heroin, cocaine). Despite this, he managed to stabilize himself psychologically, complete an apprenticeship as a mechanic, and continue his education to become an engineer. He also entered a stable relationship with a woman with whom he has a child.

A traumatic separation from his wife occurred in 2010. In the same year, he was diagnosed with chronic lymphocytic leukemia, which was treated with chemotherapy, which was discontinued prematurely after three treatment cycles in 2011 because of hepatotoxicity. In the subsequent years, the patient developed a diffuse painful polyneuropathy.

After the separation the patient became mentally destabilized. He was no longer able to work. He later became a disability pensioner. In 2012, he started permanent psychotherapeutic treatment, followed by two 3-month psychiatric/psychosomatic hospitalizations in 2013 and 2016, where he was diagnosed for continuous severe depression and complex posttraumatic stress disorder.

In 2020, he was diagnosed with metastatic colon carcinoma, which was treated with surgery and chemotherapy. This again intensified the neuropathy symptoms. Despite extensive polypharmacological treatment with paracetamol, ibuprofen, antiepileptics (pregabalin, carbamazepine), antidepressants (sertraline, escitalopram, trimipramine), sedative neuroleptics (quetiapine), and benzodiazepines (lorazepam), there was no improvement of the polyneuropathy. The patient also suffered from recurring pain in the lower sacral region following a vertebral disc herniation. This pain responded to treatment with analgesics and/or local infiltrations with cortisol and local anesthetics.

## Treatment

### Participation in study

**Figure 1:**
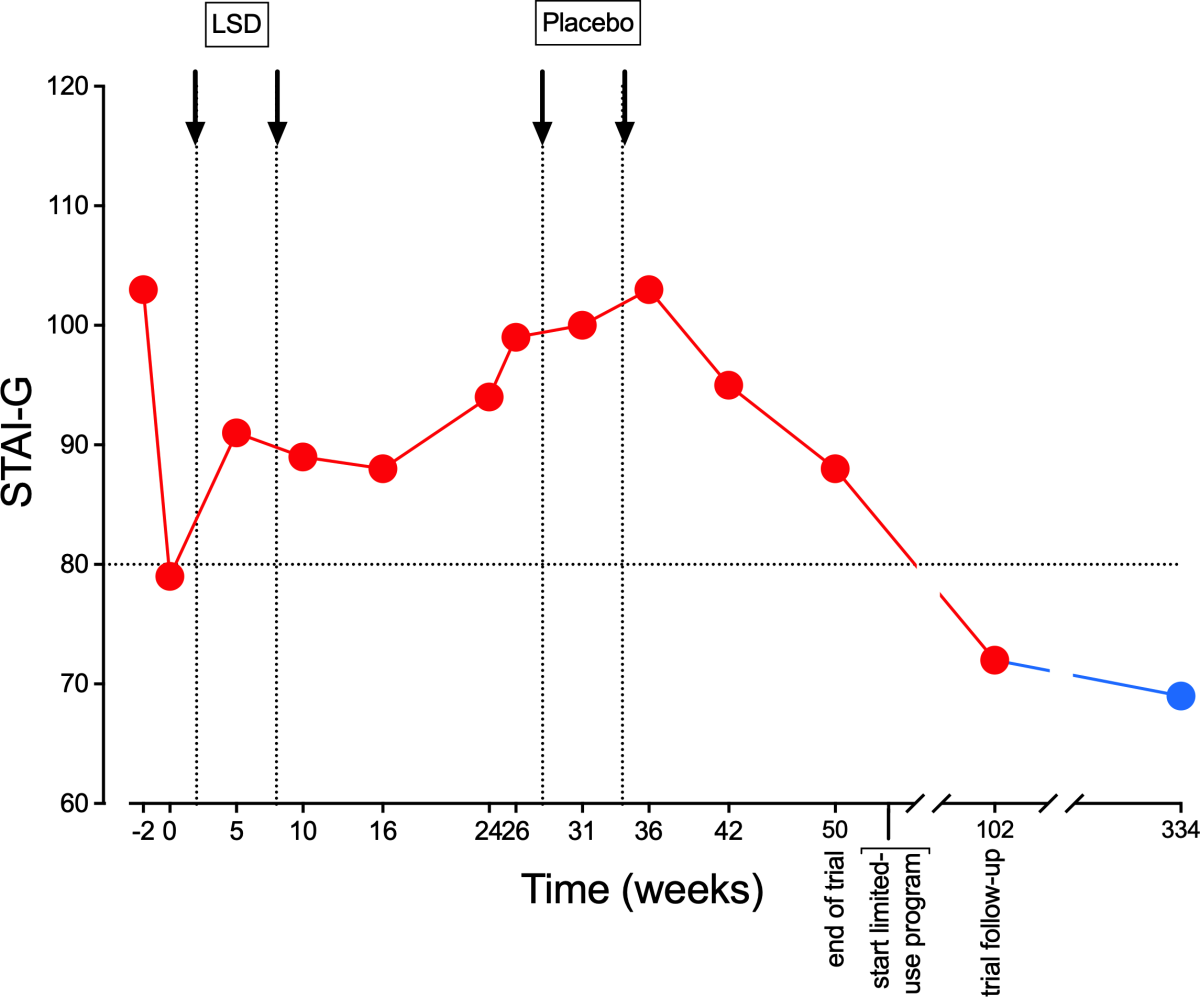
Non-response to LSD on the Spielberger State and Trait Anxiety Inventory (STAI). Values represent repeated ratings on the STAI-Global scale, which is the sum of STAI-State and STAI-Trait ratings. Scores of > 80 were needed for study inclusion, representing significant anxiety. The red dots are measures during the study. The blue dot is a measure on June 14, 2024, 334 weeks after start of the study, after 10 LSD and three MDMA dosing sessions during the limited medical use treatment phase that is described in the present report.

The patient registered as a participant in a study of LSD in patients with anxiety-related life-threatening illness (5, 11) in January 2018. He underwent two sessions with 200 µg LSD each. During the study treatments, which he completed in March 2019, he did not experience any subjective or objective LSD-typical effects. Subjective effect ratings on the 5-Dimensions of Altered States of Consciousness (5D-ASC) rating scale were the following (average of both sessions): 5D-ASC total score = 2.2% of a possible maximum score of 100%; 3D-ASC total score = 2.8/100%. Such low subjective effect scores (< 10% of the total 5D-ASC score) were only seen in two patients of the total of 40 patients who received LSD in the study. **Figure 1** presents the course of the measurement of anxiety symptoms using the Spielberger State and Trait Anxiety Inventory (STAI), showing that LSD had no anxiety-reducing effect in that patient.

### Further limited medical treatment

After the end of the study, participants who had an insufficient response were allowed to obtain limited medical use treatment with LSD and MDMA with authorization from the Swiss Federal Office of Public Health (FOPH). Authorization for the limited medical use of LSD in this patient was granted in February 2019. Throughout this treatment, STAI ratings fell permanently to values below 80, which is defined as the threshold for relevant anxiety symptoms. Between May 2019 and May 2024, the patient underwent a total of 10 dosing sessions with LSD (250-400 µg), followed by three dosing sessions with MDMA (150-175 mg). All of these experiences occurred in a group setting (group size = 5-8 participants with two therapists, including preparation and integration sessions within the group). Patients were asked to write an experience report after each treatment session. These diary-like reports had no formal requirements and served to improve the memorability and integration of psychedelic experiences and help reestablish the connection to the psychedelic experience in subsequent individual psychotherapeutic sessions. **Table 1** summarizes statements from these reports that were important for the therapeutic process and assigns them to therapy-relevant topics. The therapy-relevant topics were defined in consultation with the patient. In the total of 13 psychedelic sessions after the LSD study, the patient increasingly had experiences that were typical of LSD and MDMA. We interpreted the non-responding phase in the LSD study as a psychological unconscious defense. During this 5 years of therapy, the patient underwent an intensive process of physical and psychological stabilization, which today allows the patient to have a significantly better quality of life. He has reentered an intimate partnership after a break of more than 10 years. He can experience feelings of love for his partner, his two grandchildren, and their parents. He no longer suffers from insomnia, which was a chronic problem in the past. His relationship to death and dying has relaxed considerably.

**Table 1:** Synopsis of quotes from individual non-structured reports written shortly after the psychedelic therapy sessions.

Substance, Dose, Date	Quote from personal report written after dosing session	Therapeutic topics						
		trust, connection with oneself and	longing for security, love and partnership	being emotionally touched	freedom from pain	trauma, parents	mistrust, detachment, control	pain
LSD 300 µg 05-02-2019	I didn't want to come into contact with anyone.						X	
	At about 4.00 pm *(i.e.after 6 hrs)* I wanted to go home.						X	
LSD 400 μg 08-29-2019	I was in control of what was happening the whole time and I was in control of the trip at all times.						X	
	I realised that my body was in a suit of armour.						X	
	What became clear was that I lacked and still lack security in all my life. ...longing for a person (woman) in whom I can have blind trust and talk openly about everything, without inhibitions and fear.	X	X			X		
LSD 400μg 11-14-2019	Something inside me is looking for trust. I feel a strong desire to be able to trust someone. I keep seeing myself as maybe a 2-year-old. At this age, I have absolute trust in everything and anything. At the same time, I see the illusion of it in my eyes.	X				X		
	I only think I have an idea of love. What is love? I would love to experience it. Unconditional love.		X					
LSD 300 μg 06-26-2020	The subject of pain also came up. I tried to find out when I was last free of pain. It was impossible for me to say when that was. The frustrating thing was that after a while I realised that I didn't even have a memory of what it was like to live without pain.							X
	During my journey I hardly noticed anyone in the group.						X	
LSD 300 μg 05-14-2021	I think I've realised that I've been putting my needs on the back burner all my life. Has the time finally come to fulfil my true nature, to make room for my needs? To be more considerate of myself? To be more me? A realisation that I have been carrying around inside me for a long time, but have never acted on.	X						
	I think, or had the feeling, that I can finally let go of something. A liberating feeling, both somatically and psychologically, that is slowly making space in my tense and cramped body. I felt this particularly strongly while dancing. A liberation from very tight shackles. The iron armour suddenly became flexible.	X		X				
LSD 300 μg 08-20-2021	I feel very comfortable and in good hands in the group.	X						
LSD 300 μg 11-05-2021	For a few hours, I felt absolutely no more pain and a cosy feeling spread through my body. A state that I savoured with gratitude.				X			
LSD 300 02-18-2022	I wished I could finally let go and immerse myself in the "other" world. I have known this strong energy to control everything since my youth, perhaps even since my childhood, and it has prevented me from savouring many emotional experiences. On the other hand, it has helped me to survive in difficult situations. I would love to be able to give free rein to my feelings at least once in my life, regardless of all norms and loss of control.	X	X			X	X	
LSD 250 ug 08-26-2022	When the trembling finally stopped, I felt increasing pain, especially in my lower back and pelvis. I often had to change my position when lying and sitting. Over time, I had the feeling that I was only experiencing pain and was no longer really aware of my body.							X
MDMA 175 mg 05-11- 2023	From about 2.00 pm to 4.00 pm I felt a pleasant sensation in my upper body. A smile spread. I also had brief moments without any pain, which was a relief.			X	X			
	Below the heart, I saw a small box that opened. All my feelings were neatly organised in it, like items of clothing. One by one, the "clothes' were hung on a line. They looked splendid and shone in the sun. Looking at them somehow made me happy. Happy to see them again. However, I didn't feel them, but I got the certainty that they were still inside me.			X				
MDMA 150mq 08- 25-2023	The pain was almost all gone and the remaining pain was very weak.				X			
	At the beginning my partner was very present and I could see her clearly in my mind's eye. I relived many wonderful moments with her. Then my great-grandmother and my grandmother were there. I saw myself at my great- grandmother's sewing table, where I always sat opposite her, and she smiled at me. With my grandmother, I realised that I never saw her upset, angry or irritated. She was always very calm and kind.	X	X	X				
MDMA 175mq 05- 03-2024	Suddenly and out of nowhere I heard a voice telling me that the story with the father was over and that I could let go. The voice repeated the same sentence several times. The repetitive sentence really did me good.					X		
Microdosing MDMA, 12.5-25 mg 10-17-2023 until 02-02- 2024	Conclusion: Since the last chemotherapy, MDMA is the first active ingredient that has brought about a marked reduction in pain sensations without any side effects. With strong painkillers I always had to take a stomach protector and the effect was not nearly as pronounced as with MDMA. For me, the effect of pain relief was and is a blessing. The daily pain is a drain on my constitution and depresses my mood. The only disadvantage is the high cost, especially if the substance is taken regularly over a long period of time. I very much hope that the case report and the study will make MDMA more accessible and that many people with chronic pain can be helped.				X			X

The reports vary from one page to three pages. The quotes here were chosen to illustrate important therapeutic topics.

He has completely stopped his antidepressants, neuroleptics, and benzodiazepines. However, pain medication is still necessary occasionally, as well as for back pain and peaks of neuropathy. If neuropathic pain becomes more severe, then he currently takes nasal ketamine two to three times monthly. The patient is not a tobacco smoker but smokes a small amount of cannabis three to four times weekly for relaxation alone at home. He does not drink alcohol and since his early twenties he does not consume other illegal substances any more.

### Microdosing with MDMA

**Figure 2:**
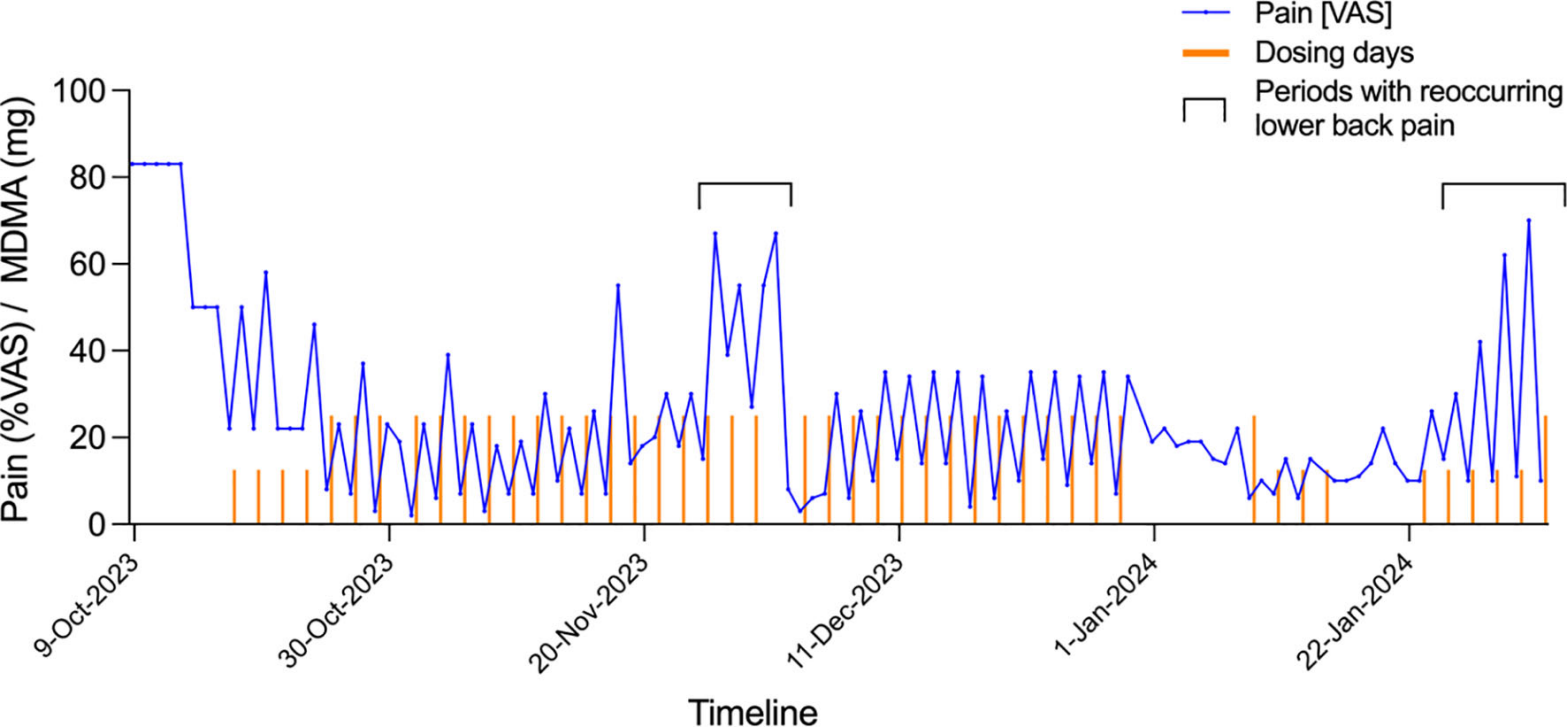
MDMA microdosing for neuralgic pain after chemotherapy. Columns shown Visual Analog Scale (VAS) scores for pain (0 = no pain, 100 = most severe pain). Small red columns indicate treatment days with 12.5 mg MDMA. Large red columns indicate treatment days with 25 mg MDMA. Treatment with 12.5 mg MDMA started on October 17, 2023. The increases in pain November 26, 2023, to December 1, 2023, and January 24, 2024, to February 1, 2024, were attributable to recurrent low back pain. This pain was treated with oral analgesics, including first paracetamol and ibuprofen and then local infiltration with cortisol and a local anesthetic.

The switch from LSD to MDMA required a new application to the FOPH, which was approved in August 2022. The reason for this change in medication was that the patient’s traumatic childhood came back into the focus of treatment, and he experienced an intensification of neuropathic pain in the tenth and last LSD session, which deterred him from having any further LSD experiences. In all three MDMA sessions, the patient reported a good pain-reducing effect, which lasted for several days after taking the drug. This prompted us to give a series of low-dose treatments to this patient. The threshold dose for a noticeable psychological effect of MDMA was assumed to be 30-40 mg. From October 9, 2023, to February 2, 2024, he took a dose of 25 or 12.5 mg MDMA every other day. These low doses of MDMA did not induce typical psychological effects of MDMA and did not interfere with daily activities. Every day, the patient recorded his pain level using a marker on a Visual Analog Scale (**Figure 2**).

Six months after the end of the MDMA microdosing treatment, the neuropathic pain disorder is still well-improved. After the 4-month trial of continuous treatment, the patient took 25 mg MDMA a few times at intervals of several weeks, and in the last 3 months he took ketamine nasal spray, 1-2 strokes of 20 mg/stroke every 1-3 weeks, instead of MDMA. This had a satisfactory pain-relieving effect. Because MDMA can trigger cardiac valve fibrosis (12, 13) we performed two cardiological examinations, including echocardiography before and after the MDMA microdosing treatment phase. Both examinations were unremarkable, and the patient did not complain of cardiac symptoms.

## Discussion

The present case report described the treatment of a patient with severe psychological stress from a traumatic childhood and traumatic separation who was undergoing oncology treatment and suffered from psychological distress from this life-threatening disease and experienced chemotherapy-induced diffuse neuropathic pain disorder. No effects of a medium dose of LSD (200 µg) were initially noticed. This made the patient one of the very few non-responders in an LSD study with regard to acute subjective effects and the therapeutic response. Only when the LSD dose was increased to a very high 400 µg dose during further treatment did the patient have an LSD-specific acute reaction. Afterward, the dose was lowered to 250 µg while still experiencing LSD-specific subjective effects. The patient underwent a series of 10 LSD sessions, in which he experienced therapeutic benefits, such as confidence building, a reduction of social anxiety, a reduction of fear of death, improvements in sleep, and a reduction of depressive symptoms. Thus, there was no subjective response and no therapeutic response during the clinical study, but subjective and therapeutic responses could be achieved later with higher doses and repeated LSD treatments outside the constraints of the clinical study and with a Swiss license for the limited medical use of psychedelics. This finding is consistent with the view that greater therapeutic responses are seen in patients who experience greater acute psychedelic effects (5). This could mean that the subjective experience causally contributes to the therapeutic process. It could also mean that patients who are able to perceive and experience emotional and self-altering effects of psychedelics might be more likely to improve, with the subjective effect a predictive biomarker of the therapeutic response. The finding also illustrates that some patients may not respond to the standard dosing of a psychedelic in a clinical trial but may respond to individualized dosing and treatment approaches. With regard to polyneuropathy that occurred during chemotherapy for cancer, the patient experienced no clear progress under LSD treatment. Thus, no analgesic effects of LSD were observed. Only when switching to MDMA-assisted therapy did the patient experience a clear improvement in pain under the acute effect up to 1 week after the single-dose administration. This clinical observation prompted a trial of MDMA microdosing (12.5-25 mg every other day for 4 months). This treatment led to a sustained improvement in neuropathic pain. To our knowledge, a pain-reducing effect of repeated low doses or “microdoses” of MDMA has not been previously reported and would need further confirmatory studies. Reductions of chronic pain have been previously reported among patients with chronic pain who were treated for posttraumatic stress disorder. However, these patients were treated with fully psychoactive doses and only three times (first 120 mg and then 180 mg twice). Thus, full doses of MDMA were used, whereas we also found reductions of pain with repeated every-other-day doses of only 12.5-25 mg that were not psychoactive and did not interfere with daily activities. How MDMA would reduce pain is unclear. It is not known as an analgesic *per se*. However, in humans, MDMA produces acute effects via the release of norepinephrine, serotonin, and oxytocin (7, 8), which are all known modulators of pain. Animal studies have also shown a possible effect on the opioid system (14). Subjectively, the patient reported that he felt more relaxed overall in everyday life, an impression that was also confirmed by his partner. Thus, MDMA may have influenced aspects of emotional pain processing and cognitive pain perception. MDMA has been shown to reduce the response to negative emotional stimuli, including lower amygdala activation in response to fear (15–18). MDMA may thus reduce negative emotional reactions to pain. It has also been shown to enhance extinction learning (19–21) and may potentially help with pain perception desensitization. MDMA may also enhance positive mood and reduce depressive symptoms, thereby reducing the negative impact of pain on mood (9) rather than primary pain perception. Certainly, diffuse neuropathic pain disorders cannot be treated with muscular relaxation alone, and relaxation that is elicited by MDMA should be seen as more than a muscular relaxation process that promotes such general factors as confidence, trust, and openness (17, 22). Importantly, MDMA continued to exert analgesic effects in the present patient, even when it was administered at doses that did not produce overt emotional effects, indicating additional beneficial effects on pain perception that may be independent of its mood-altering properties.

In conclusion, the present description of an improvement in a neuropathic pain disorder with MDMA is one isolated case that is described in the context of a complex disease state. The mechanisms that led to improvements are largely unexplained. Further observations and ultimately controlled studies are necessary to shed more light on the potential of MDMA in chronic pain disorders.

## Data Availability

The original contributions presented in the study are included in the article/supplementary material. Further inquiries can be directed to the corresponding author.
